# Supplementary material for: Model-Informed Target Morning 17α-Hydroxyprogesterone Concentrations in Dried Blood Spots for Pediatric Congenital Adrenal Hyperplasia Patients
Source: Pharmaceuticals (Basel). 2023 Mar 21;16(3):464. doi: 10.3390/ph16030464 (PMC10051286; doi:10.3390/ph16030464)
Supplement: Supplementary file 1 [file pharmaceuticals-16-00464-s001.zip › pharmaceuticals-2254452-supplementary.pdf]

pharmaceuticals

Deriving target morning 17α-hydroxyprogesterone concentrations in dried blood spots for pediatric congenital adrenal hyperplasia patients

Viktoria Stachanow [1,2], Uta Neumann [3], Oliver Blankenstein [3,4], Nele Alder-Baerens [4], Davide Bindellini [1,2], Peter Hindmarsh [5], Richard Ross [6], Martin J Whitaker [6], Johanna Melin [1,2], Wilhelm Huisinga [7], Robin Michelet [1,*] and Charlotte Kloft [1]

[1] Department of Clinical Pharmacy and Biochemistry, Institute of Pharmacy, Freie Universitaet Berlin, Kelchstr 31, 12169 Berlin, Germany; viktoria.stachanow@fu-berlin.de (V.S.); davide.bindellini@fu-berlin.de (D.B.); johanna.s.melin@gmail.com (J.M.); charlotte.kloft@fu-berlin.de (C.K.)
[2] Graduate Research Training Program, PharMetrX, 12169 Berlin, Germany
[3] Charité-Universitätsmedizin, Freie Universität Berlin, 13353 Berlin, Germany; uta.neumann@charite.de (U.N.)
[4] Labor Berlin, Charité Vivantes GmbH,13353 Berlin, Germany; nele.alder-baerens@charite.de
[5] Developmental Endocrinology Research Group, UCL Institute of Child Health, London WC1E 6BT, UK
[6] Department of Oncology and Metabolism, University of Sheffield, Sheffield S10 2TN, UK; r.j.ross@sheffield.ac.uk (R.J.R.); martin.whitaker@sheffield.ac.uk (M.J.W.)
[7] Institute of Mathematics, Universität Potsdam, 14476 Potsdam, Germany; huisinga@uni-potsdam.de
* Correspondence: robin.michelet@fu-berlin.de

1 Pharmacokinetic/Pharmacodynamic model structure

Differential equations

$$dA_{depot}/dt = -\frac{V_{abs,max} \cdot A_{depot}}{K_{abs,50} + A_{depot}}$$

$$dA_{pla,child}/dt = \frac{V_{abs,max} \cdot A_{depot}}{K_{abs,50} + A_{depot}} - CL/V_{cen} \cdot A_u - Q/V_{cen} \cdot A_u + Q/V_{per} \cdot A_{per}$$

$$dA_{per}/dt = Q/V_{cen} \cdot A_u - Q/V_{per} \cdot A_{per}$$

$$dA_{RBC}/dt = K_{aRBC} \cdot A_u - K_{aRBC} \cdot A_{RBC}$$

$$dC_{17-OHP,DBS}/_{dt} = \mathrm{k_{syn}} \cdot \mathrm{I} - \mathrm{k_{deg}} \cdot C_{17-OHP,DBS}$$

Initial conditions

$$A_{depot,0} = F * DOSE$$

$$A_{pla,child,0} = BASE_{child,pla} * V_{cen}$$

$$A_{per,0} = 0$$

$$A_{RBC,0} = 0$$

$$C_{17-OHP,DBS,0} = OHP_{BASE}$$

State variables and outputs

$$\mathrm{k_{syn}} = OHP_{BASE} \cdot \mathrm{k_{deg}}$$

$$\mathrm{I} = 1 - \frac{I_{max} \cdot C_{pla,child}{}^{Hill}}{IC_{50}{}^{Hill} + C_{pla,child}{}^{Hill}}$$

$$K_{dA} = K_d * V_{cen}$$

$$A_{max} = B_{max} * V_{cen}$$

$$A_u = \frac{A_{pla,child} - K_{dA} \cdot (1 + NS_{Alb}) - A_{max} + \sqrt{(A_{pla,child} - K_{dA} \cdot (1 + NS_{Alb}) - A_{max})^2 + 4 \cdot A_{pla,child} \cdot K_{dA} \cdot (1 + NS_{Alb})}}{2 \cdot (1 + NS_{Alb})}$$

$$C_{pla,child}(t) = {A_{pla,child}(t)}/{V_{cen}}$$

Estimated model parameters

$k_{deg,ind} = k_{deg,pop} \cdot e^{\eta_{kdeg,ind}}$ $\eta_{kdeg} \sim N(0, \omega_{kdeg})$

$IC_{50,ind} = IC_{50,pop} \cdot e^{\eta_{IC50,ind}}$ $\eta_{IC50} \sim N(0, \omega_{IC50})$

Fixed model parameters*

$Hill = 1$

$OHP_{BASE,pop} = OHP_{BASE,observed}\ OR\ {}^{1}/_{2}\, LLOQ$

$OHP_{BASE,ind} = OHP_{BASE,pop} \cdot e^{\eta_{OHPBASE,ind}} \cdot RUV$ $\eta_{OHPBASE} \sim N(0, \omega_{OHPBASE})$

$\eta_{OHPBASE} = 1$

*2 out of 18 17-OHP baseline DBS observations were below the lower limit of quantification (LLOQ) and were replaced by the LLOQ divided by two (= 1.3/2 nmol/L).

Handling data below the lower limit of quantification

25% (22/88) of all venous DBS 17-OHP concentrations were below the LLOQ (22.5% in young children and 35.3% in infants). Thus, the M3 method [20] was applied where all observations were fitted to the model, including the concentrations below the LLOQ which were treated as censored observations for which the likelihood of an observation being below the LLOQ given the model parameters is maximized.

2 NONMEM model code

```
$PROBLEM
PKPD indirect response model, cortisol plasma and 17-OHP in DBS, pediatric model

$INPUT
ID TIME AMT DROP=RATE MDV ODV EVID BLQ CMT FLAG ICL IV1 IQ IV2 IKaRBC IIBASE
IBMAX IKD INSALB IVM IKM IF1

$DATA  Data_PKPD.csv IGNORE=@
$SUBROUTINE  ADVAN13 TOL=15

$MODEL
NCOMPARTMENTS=5 NPARAMETERS=3
COMP = (DEPOT DEFDOSE)               ;dose compartment
COMP = (CENTRAL DEFOBSERVATION)      ;central plasma concentration
COMP = (PERIPH)                      ;peripheral plasma compartment
COMP = (OUTPUT1)                ;compartment with cortisol bound to erythrocytes
COMP = (EFFECT)                 ;compartment with indirect response, inhibition of synthesis

$PK
;; individual PK parameter estimates from previous PK model (Stachanow et al., 2022) [11]

CL = ICL                ;clearance (CL)
V1 = IV1                ;central volume of distribution (Vcen)
Q = IQ                  ;intercompartmental clearance (Q)
V2 = IV2                ;peripheral volume of distribution (Vper)
KaRBC = IKaRBC          ;association constant for red blood cells (kaRBC)
IBASE = IIBASE          ;pediatric plasma cortisol baseline (BASEchild,pla)
BMAX = IBMAX            ;maximum binding capacity for CBG binding (Bmax)
KD = IKD                ;equilibrium dissociation constant for CBG binding (Kd)
NSALB = INSALB          ;linear non-specific parameter for albumin binding (NSAlb)
VM = IVM                ;maximum absorption rate (Vmax)
KM = IKM                ;amount in depot compartment resulting in half of Vmax (Km)
F1 = IF1                ;bioavailability (F)

;; PD Parameters
IMAX  = 1               ;maximum inhibitory effect (Imax)
GAM   = 1               ;Hill coefficient (Hill)


TVKDEG  = THETA(1)      ;first-order degradation rate constant of 17-OHP (kdeg)
TVIC50  = THETA(2)      ;cortisol conc. leading to 50% of the maximum inhibitory effect (IC50)
```

```
MU_1  = TVKDEG
MU_2  = TVIC50

KDEG  = EXP(MU_1 + ETA(1))
IC50  = EXP(MU_2 + ETA(2))

;17-OHP baseline observations in DBS
BASEDV = ODV
;17-OHP baseline observations in DBS if no baseline observation given
IF(ODV.LT.1.3) THEN
BASEDV = 0.65                     ;BLQ baselines replaced by 1/2 LLOQ
ENDIF

OHPBASE = BASEDV*EXP(ETA(3)*THETA(3))

KSYN = OHPBASE*KDEG               ;17-OHP synthesis rate (ksyn)

;converting concentrations to amounts
KDa = KD*V1
AMAX = BMAX*V1

;amount at timepoint 0 in central plasma compartment and in 17-OHP DBS compartment
A_0(2) = IBASE*V1
A_0(5) = OHPBASE

IF(IC50.EQ.0) THEN
IC50 = 0.001
ENDIF
IF(IC50.LT.0) THEN
IC50 = 0.001
ENDIF

k10 = CL/V1
k12 = Q/V1
k21 = Q/V2

; Time after dose
TAD = 0
IF (AMT.GT.0) THEN
TDOS = TIME
TAD = 0.0
ENDIF
IF (AMT.EQ.0) TAD = TIME-TDOS
IF (TAD.LT.0) TAD = 0
```

```
;;LLOQ of 17-OHP in DBS
LLOQ = LOG(1.3)

$DES
;;calculating unbound amount in plasma with binding model of previous model(s)
AUP = (A(2)-KDa*(1+NSALB) – AMAX + SQRT(((A(2) - KDa*(1 + NSALB) -AMAX)**2) +
4*KDa*A(2)*(1 + NSALB)))/(2*(1 + NSALB))

DADT(1) = -(VM*A(1)/(KM+A(1)))                                          ;saturable absorption
DADT(2) =  (VM*A(1)/(KM+A(1))) - k10*AUP - k12*AUP + k21*A(3)           ;central compartment
DADT(3) = -k21*A(3)+k12*AUP                                             ;peripheral compartment
DADT(4) = AUP* KaRBC -A(4)* KaRBC                                 ;red blood cell compartment

CP = A(2)/V1
IF(CP.LT.0) THEN
CP=0.001
ENDIF

INH = 1-(IMAX*CP**GAM/(IC50**GAM+CP**GAM))
DADT(5) = KSYN*INH-KDEG*A(5)                                      ;17-OHP compartment


$ERROR
IPRED = A(5)

IF(IPRED.GT.0) THEN
IPRED = IPRED
ELSE
IPRED = IPRED+0.0001
ENDIF

IPRED = LOG(IPRED)
W = THETA(3)
IRES= DV-IPRED
IWRES = IRES/W

IF(BLQ.EQ.0) THEN
F_FLAG = 0
Y = IPRED+W*EPS(1)

ELSE
F_FLAG = 1
DUM = (LLOQ-IPRED)/W
CUMD = PHI(DUM)
```

```
Y = CUMD
MDVRES = 1
ENDIF
$THETAI
THETA(1:2 )= DLOG(THETAI(1:2))

$THETAR
THETAR(1:2) = EXP(THETA(1:2))

$THETA
(0, 1.22)          ;1. KDEG [1/h]
(0, 20)            ;2. IC50 [nmol/L]]
(0, 0.2)           ;3. RUV

$OMEGA
0.0025 FIX         ;KDEG
(0.0025, 0.5)      ;IC50
1 FIX              ;OHPBASE

$SIGMA 1 FIX

$ESTIMATION METHOD=IMPMAP LAPLACE INTERACTION NITER=2000 ISAMPLE=300
SEED=987213 NOABORT NSIG=3 PRINT=1 NOABORT FILE=psn.ext
SIGL=9 GRD=DDDS
$ESTIMATION METHOD=IMPMAP LAPLACE INTERACTION NITER=200 ISAMPLE=3000
SEED=987213 NOABORT NSIG=3 PRINT=1 NOABORT FILE=psn.ext
SIGL=15 EONLY=1 GRD=DDDS
$COVARIANCE PRINT=E UNCONDITIONAL SIGL=9

$TABLE ID TIME TAD DV PRED IPRED WRES IWRES CWRES MDV BLQ AUP CP FLAG
ONEHEADER NOPRINT FILE=sdtab313_1_2

$TABLE ID TIME TAD DV CL V1 Q V2 KaRBC IBASE BMAX KD NSALB VM KM F1 ODV
FLAG ETA1 ETA2 ETA3 KSYN KDEG OHPBASE IMAX IC50 GAM NOAPPEND ONEHEADER
NOPRINT FILE=patab313_1_2

$TABLE ID TIME TAD DV FLAG NOAPPEND ONEHEADER NOPRINT FILE=catab313_1_2
$TABLE ID TIME TAD DV ODV FLAG NOAPPEND ONEHEADER NOPRINT FILE=cotab313_1_2
```

3 Supplementary Tables and Figures

Table S1. Summary of population characteristics in all datasets leveraged in the modeling and simulation analysis, by framework step as described in Figure S1.

	Step A: PK/PD modeling	Step B: Bland-Altman and Passing-Bablok analysis	Step C: Simulation of target range
**Population**	Pediatric CAH patients (n=18), clinical study	Pediatric CAH patients (n=15), clinical routine monitoring	Non-CAH children (n=28)
**Analyte and matrix**	Cortisol in plasma<br>17-OHP in venous DBS	Cortisol and 17-OHP in capillary and venous DBS	Cortisol in plasma
**Number of samples**	88 (per matrix)	15	2016
**Age, median (range)**	2.5 years<br>(4 months – 5 years)	8 years<br>(2 months – 11 years)	(5-9) years
**Weight, median (range)**	15 (7-21) kg	-	-

Congenital adrenal hyperplasia (CAH), 17α-hydroxyprogesterone (17-OHP), dried blood spots (DBS)

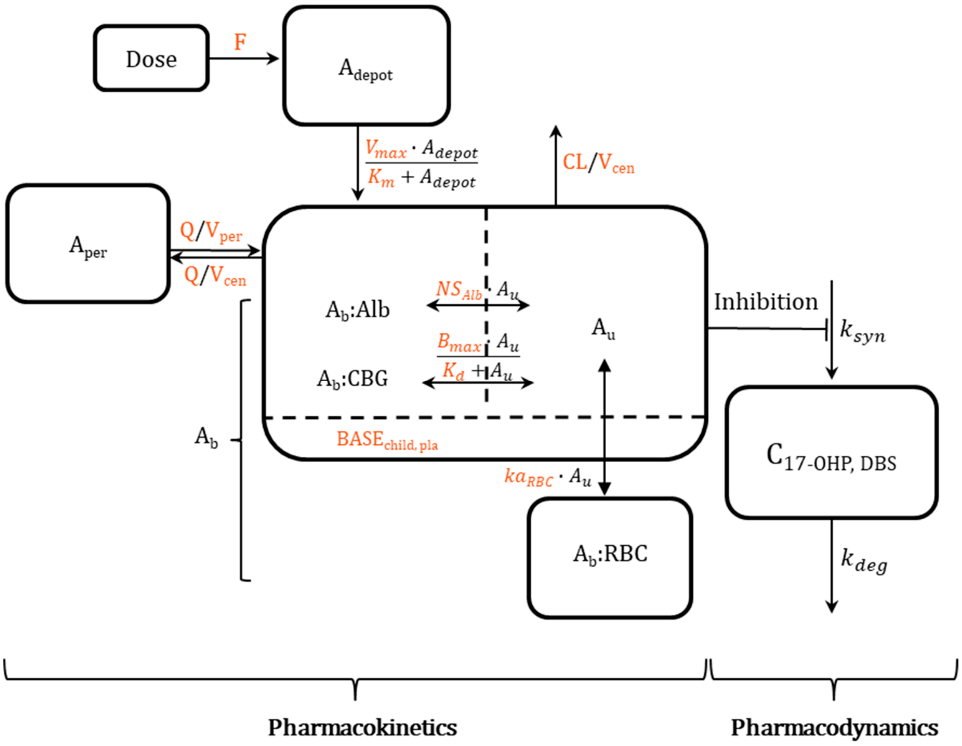

Figure S1. Schematic representation of developed pharmacokinetic/pharmacodynamic (PK/PD) model. PK parameters of which the pediatric individual estimates from the previous PK model (Stachanow et al.) [11] were used as part of the dataset are marked in orange.

Pharmacokinetics: Bioavailability (F), amount in depot compartment (A_{depot}), maximum absorption rate (V_{max}), amount in depot compartment resulting in half of V_{max} (K_m), amount bound (A_b), amount bound to albumin (A_b:Alb), amount associated to red blood cells (A_b:RBC), unbound amount (A_u), amount bound to corticosteroid-binding globulin (A_b:CBG), linear non-specific parameter for albumin binding (NS_{Alb}) and association to red blood cells (ka_{RBC}), maximum binding capacity (B_{max}), equilibrium dissociation constant (K_d), intercompartmental clearance (Q), central volume of distribution (V_{cen}), peripheral volume of distribution (V_{per}), cortisol plasma baseline of children ($BASE_{child, pla}$). The dashed line divides the central compartment into the A_b and A_u subcompartments, respectively.

Pharmacodynamics: 17α-hydroxyprogesterone (17-OHP) concentration in dried blood spots ($C_{17\text{-OHP, DBS}}$), synthesis rate constant of 17-OHP (k_{syn}), first-order elimination rate constant of 17-OHP (k_{deg}).

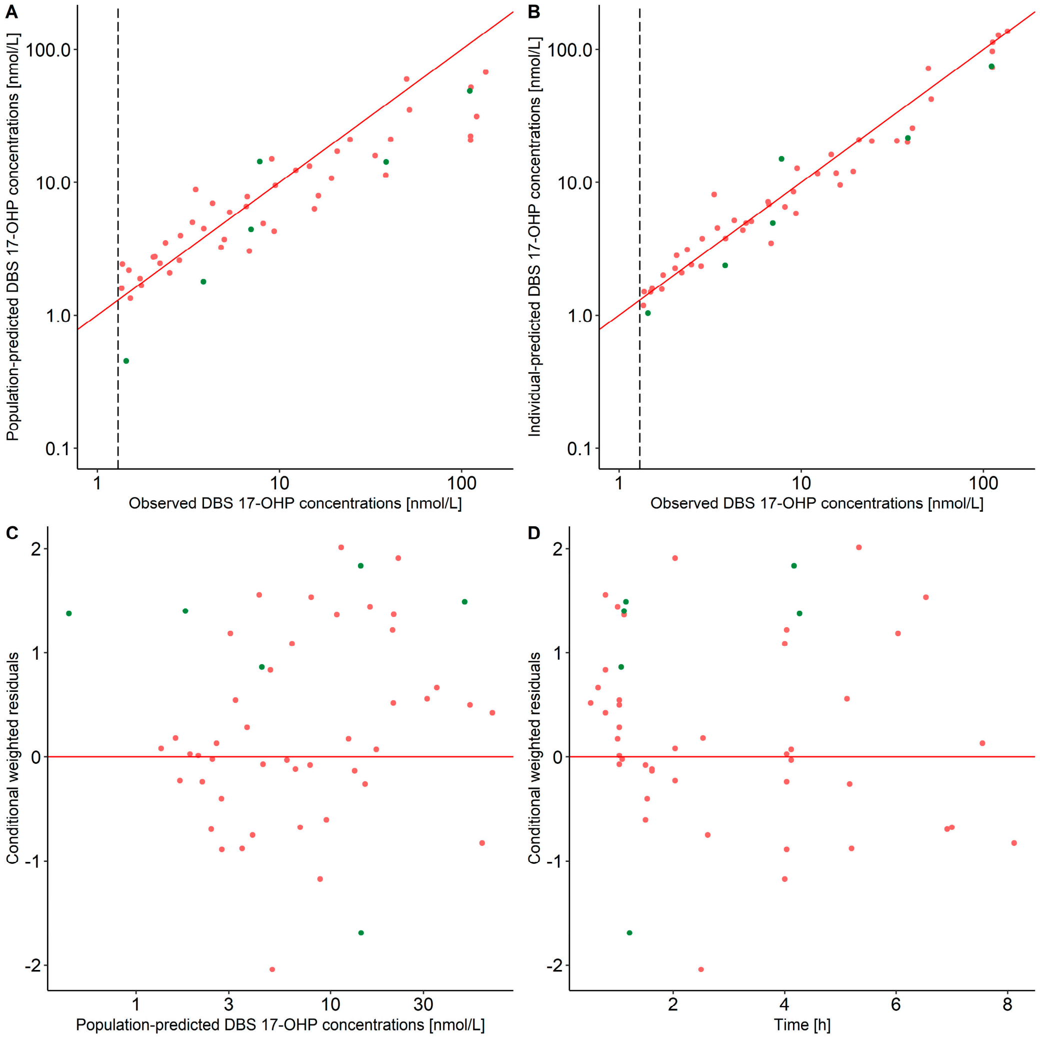

Figure S2. Goodness-of-fit plots for developed pharmacokinetic/pharmacodynamic (PK/PD) model. A: Population-predicted dried blood spot (DBS) 17α-hydroxyprogesterone (17-OHP) concentrations versus observed DBS 17-OHP concentrations, B: Individual DBS 17-OHP predictions versus observed DBS 17-OHP concentrations, C: Conditional weighted residuals versus population-predicted DBS 17-OHP concentrations, D: Conditional weighted residuals versus time. Red dots: children (cohort 1, age: 2-6 years), green dots: infants (cohort 2, age: 28 days-2 years), red line: line of identity (A, B), line y=0 (C, D), vertical dashed line: lower limit of quantification (LLOQ) = 1.3 nmol/L.

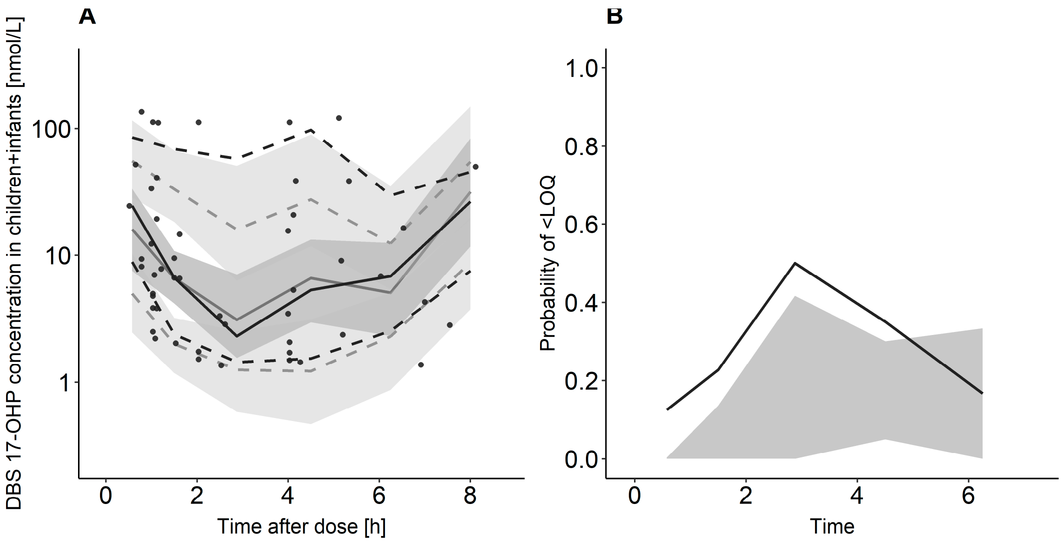

Figure S3. Visual predictive check (n=1000) for developed pharmacokinetic/pharmacodynamic (PK/PD) model.
A: Circles: 17α-hydroxyprogesterone (17-OHP) dried blood spot (DBS) observations, solid line: 50th percentile of observed (black) and simulated (gray) DBS 17-OHP concentrations, dashed lines: 10th and 90th percentiles of observed (black) and simulated (gray) DBS 17-OHP concentrations, shaded areas: 95 % confidence intervals for the percentiles of the simulated data.
B: Black line: Observed probability of DBS 17-OHP concentrations below lower limit of quantification (LLOQ), gray area: 95 % confidence interval for simulated probability of 17-OHP concentrations below LLOQ.

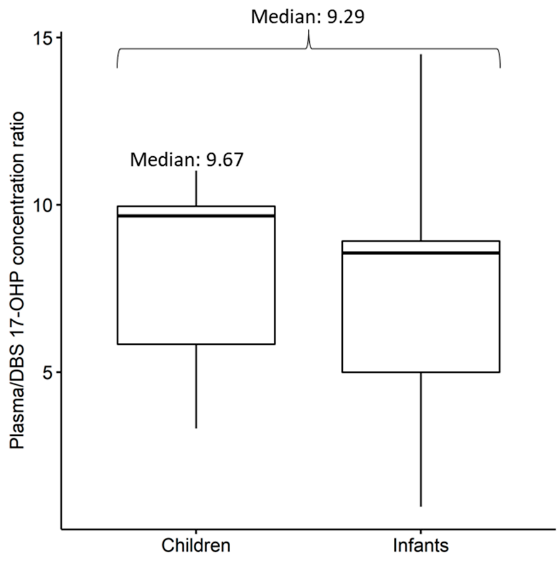

Figure S4. Plasma to dried blood spot (DBS) 17α-hydroxyprogesterone (17-OHP) concentration ratio, measured at baseline in the morning, in young children and infants.
